# Multifunctional Nanoplatforms as a Novel Effective Approach in Photodynamic Therapy and Chemotherapy, to Overcome Multidrug Resistance in Cancer

**DOI:** 10.3390/pharmaceutics14051075

**Published:** 2022-05-17

**Authors:** Martin Majerník, Rastislav Jendželovský, Jana Vargová, Zuzana Jendželovská, Peter Fedoročko

**Affiliations:** Institute of Biology and Ecology, Faculty of Science, Pavol Jozef Šafárik University in Košice, Šrobárova 2, 041 54 Košice, Slovakia; martin.majernik@upjs.sk (M.M.); jana.vargova@upjs.sk (J.V.); zuzana.jendzelovska@upjs.sk (Z.J.); peter.fedorocko@upjs.sk (P.F.)

**Keywords:** photodynamic therapy, multidrug resistance, multifunctional nanoplatforms, therapeutic synergism, chemotherapy

## Abstract

It is more than sixty years since the era of modern photodynamic therapy (PDT) for cancer began. Enhanced selectivity for malignant cells with a reduced selectivity for non-malignant cells and good biocompatibility along with the limited occurrence of side effects are considered to be the most significant advantages of PDT in comparison with conventional therapeutic approaches, e.g., chemotherapy. The phenomenon of multidrug resistance, which is associated with drug efflux transporters, was originally identified in relation to the application of chemotherapy. Unfortunately, over the last thirty years, numerous papers have shown that many photosensitizers are the substrates of efflux transporters, significantly restricting the effectiveness of PDT. The concept of a dynamic nanoplatform offers a possible solution to minimize the multidrug resistance effect in cells affected by PDT. Indeed, recent findings have shown that the utilization of nanoparticles could significantly enhance the therapeutic efficacy of PDT. Additionally, multifunctional nanoplatforms could induce the synergistic effect of combined treatment regimens, such as PDT with chemotherapy. Moreover, the surface modifications that are associated with nanoparticle functionalization significantly improve the target potential of PDT or chemo-PDT in multidrug resistant and cancer stem cells.

## 1. Introduction

Cancer is the first or second leading cause of death before the age of 70 years in the majority of countries worldwide [1]. The incidence and mortality of cancer is affected by multiple factors, including lifestyle, the type of cancer and its specificity, stage of cancer, mode of treatment, etc. [2,3]. Thus, the efficacy of treatment varies and the need for personalized therapy [4], the development of a novel therapy development [5,6,7,8], and the search for novel anticancer drugs [9] play a crucial role. One of the most serious problems that significantly reduces the therapeutic effectivity in cancer treatment is the phenomenon of multidrug resistance (MDR). The theory of MDR is not novel; initially it was prevalently associated with the reduction of chemotherapy efficacy [10] but it is now widely known that the phenomenon of MDR can also significantly reduce the therapeutic effectivity of other treatment approaches, even photodynamic therapy (PDT) [11]. The mechanism of MDR is largely associated with the ATP-binding cassette (ABC) transporters [12] with broad substrate specificity, which includes many therapeutics and photosensitizers (PSs), too [13,14,15,16,17,18,19,20,21,22,23]. Therefore, restricting substrate specificity and bypassing the efflux of target agents represent one of the possible solutions for limiting MDR. The concept of a dynamic nanoplatform using non-biodegradable nanoparticles (NPs) to permanently retain PSs has been established on just this base and it has been progressively developed in the last fifteen years [24,25,26,27,28,29,30,31,32,33,34,35,36]. When we talk about nanotechnology, we consider a scale—an order of magnitude—of size, or length. The prefix ‘nano-’ is derived from the Greek word *nannos*, meaning “very short man”. In scientific units ‘nano’ is used to denote one-billionth of the base unit. Nanotechnology includes the formation and use of materials, structures, devices, and systems that have unique properties because of their small size [37]. The term ‘nanotechnology’ can be dated back to 1974 when it was first used by Norio Taniguschi. Taniguschi described nanotechnology as the technology that forms materials at the nanometer level [38]. Nanomaterials and NPs, the nanometer-sized objects, are the leading edge of the rapidly developing field of nanotechnology and have great applicability in biology and medicine. As NPs are much smaller in size than the cells of living organisms, they are suitable for bio tagging and labeling, drug or gene delivery, diagnosis and detection of specific proteins or pathogens, etc. In general, simple NPs are made from a single material, whereas composite and core/shell NPs are composed of two or more materials. The core itself can consist of several functional layers, allowing the use of nanomaterials in multifunctional approaches. The core particle is usually surrounded and protected by another outer layer or by several layers (a shell) that are composed of some inert material, organic molecules, or biocompatible materials. However, specific linker molecules, ligands, and additional layers are more often conjugated on the surface of NPs in order to improve and add some useful properties, and to increase the biocompatibility of the nanomaterial [39,40]. Technological progress makes it possible to create novel materials, modify the characteristics of currently created materials, or prepare some multimaterial structures. Novel technologies enable multifunctional nanoplatforms to be constructed with enhanced targeting to the particular sites of the tumor mass. In 2015, Yang et al. [41] were the first to introduce multifunctional chemo-PDT and fluorescent imaging systems based on mesoporous silica NPs. Subsequently, many types of NPs and PSs were analyzed for the purpose of improving the therapeutic efficacy of PDT and chemotherapy, not only against the multidrug resistant cancer cells [41,42,43,44,45,46,47,48,49,50,51,52,53,54,55,56,57], but also against the cancer stem cell phenotype [58,59,60,61].

In the presented paper, we describe and summarize the role and applicability of NPs in the improvement of tumor treatment, mainly in relation to PDT or PDT and chemotherapy. We have not focused on the huge variety of cancer aspects; on the contrary, our effort was dominantly aimed at the phenomenon of MDR. Based on the presented facts, we assume that the application of multifunctional nanoplatforms could represent a potential solution for restricting MDR in tumors affected by PDT and improve the applicability of PDT in cancer treatment, as a monotherapy or in combination with chemotherapy.

## 2. Nanoparticles—General Systematization

Over the last twenty years, great progress has been made in the field of NP development and their utilization can be found in a huge number of therapeutic approaches [62,63]. Generally, NPs are defined as submicroscopic particles with a size range from 1 to 100 nm [64]. Many refined review papers discussing the systematic classification, description of preparation methods, and their complex physical and biochemical characterization of NPs have been published [64,65]. However, there are several important applications of nanomaterials, and there is no doubt that material engineering represents one of the most progressive scientific areas. The development of novel materials is also substantial [47,48,66,67,68], and the validity and completeness of any systematic nomenclature related to the systematization of NPs is therefore temporary. Thus, the detailed and extensive description of NPs’ systematization, synthesis, and structure is not included in this paper.

Generally, NPs are naturally occurring or chemically prepared synthetic materials. Initially, NPs are very often categorized as active or passive; being active means that they carry active surface moieties [69,70,71].

Lucky et al. (2015) presented a classification system based on the functions or tasks of NPs, namely in PDT. According to the system, NPs are divided into three classes: carriers of PSs, PSs by themselves, and energy transducers of PSs. Currently, the first class of NPs, having the role of PS carriers, is composed of biodegradable and non-biodegradable NPs. The group of biodegradable NPs is represented by polyester and polyacrylamide NPs, liposomal NPs, dendrimer-based NPs, and natural macromolecule-based NPs that are presented by albumin. The class of nonbiodegradable NPs is composed of silica, gold, and magnetic NPs.

### 2.1. The First Class of NPs—Carriers of PSs

Retrospectively, the greatest potential of NPs was originally seen in their delivery role. For this purpose, they were firstly used as a vaccine delivery vehicle, focusing on a slow and controlled cargo release [72]. This represented a crucial milestone, which enabled for the intravenous application of solid NPs without the risk of embolism [64]. NPs were then utilized for a variety of therapeutic interventions; however, PDT and chemotherapy have been studied most extensively. At present, there is no doubt that chemotherapy is a very important method in cancer treatment. Nonetheless, one of its major drawbacks is its non-specificity, the resulting systemic toxicity, and the presence of side effects [73]. The utilization of NPs as delivery vehicles to specific targets is expected to solve this problem. As well as reducing side effects, the bioavailability, solubility, biodistribution, reduction of drug resistance, and nonspecific toxicity could be improved [74,75,76]. Therefore, nano-oncology is currently one of the most extensively studied area of nanotechnology.

Biodegradable NPs have been very intensively studied as nano-vehicles, also being suitable for application in PDT. Works with liposomes initially started in the mid-1960s, with Alec Bangham and colleagues observing the bimolecular leaflet membrane structures formed by adding water to dry phospholipids [77]. Based on their similarity to cell membranes, liposomes were recognized as the conventional model for a biophysical analysis of cell membranes [78]. Subsequently, the main role of liposomes was altered to that of a tool for the study of a drug delivery system, which was later confirmed by several in vivo analyses [79,80,81].

The analyses of liposomes as the most extensively studied biodegradable NPs for PDT had already started in the 1980s. The majority of PSs show a typical hydrophobic nature, which is related to the chemical structure of the PS molecules. Due to their low water solubility, the molecules of PSs are not in monomeric form, but rather form aggregates in aqueous environments. Consequently, this behavior strongly limits the photoactive properties of PS molecules, as their monomeric form is a fundamental condition for reactive oxygen species (ROS) generation [82]. For this reason, hydrophobic PSs can be simply formulated in organic solvents; the most common solvent is dimethyl sulfoxide (DMSO) [83]. Nevertheless, DMSO has been reported to interfere with cell-based assays, to inhibit the cytotoxicity of some drugs, e.g., platinum, and last, but not least, to cause adverse effects in humans, such as gastrointestinal and skin reactions [84,85,86]. Therefore, the wider use of DMSO for in vivo or a clinical application is controversial. Several transport systems [87,88,89,90,91,92,93,94] or solvents [83] have been investigated to improve the low water solubility of hydrophobic drugs.

**Figure 1:**
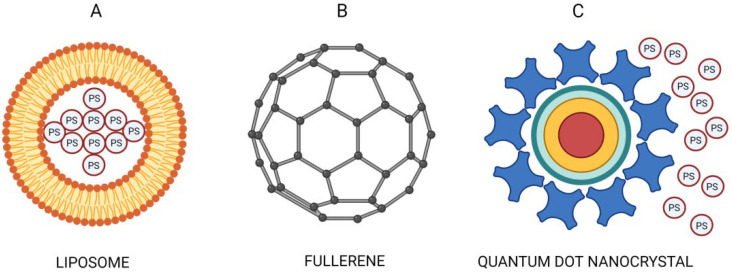
(**A**) Liposomes as a representative of the first class of NPs—carriers of PSs. (**B**) Fullerene as a representative of the second class of NPs—PSs by themselves and (**C**) Quantum dot nanocrystal as a representative of the third class of NPs—energy transducers of PSs.

The utilization of liposomes represents one of the possible approaches to deliver hydrophobic agents (see Figure 1A). Liposomes are membrane structures that are composed of lipid molecules, and their composition makes it possible to incorporate the hydrophobic agents within the lamellar structures, whereas hydrophilic agents could be loaded in an aqueous core [95]. Currently, some PSs that have been prepared in liposomal formulations are officially approved for clinical utilization; verteporfin, trade name Visudyne (a benzoporphyrin derivative monoacid (BPD-MA in liposomal formulation), and temoporfin, trade name Foscan (meso-tetra-hydroxyphenyl-chlorin (mTHPC) in liposomal formulation) seem to be the best known representatives [96]. Visudyne is used to eliminate abnormal blood vessels in the eye, which is linked to the wet form of macular degeneration, and Foscan is used to treat squamous cell carcinoma of the head and neck [96]. Besides the fact that these liposomes show good biocompatibility, the encapsulation efficiency is still relatively low, and improvement of the delivery efficiency of liposomes is therefore fundamental [97]. Moreover, premature release of the PS and susceptibility to opsonization can noticeably lower the treatment efficiency of this therapeutic modality [98,99]. For that reason, liposomes are not optimal PS carriers, and further structural modification [57] and development were therefore undertaken.

### 2.2. The Second Class of NPs—PSs by Themselves

After the drug carriers, the second class of NPs is represented by some nanoscale materials that are capable of generating ROS, where interaction or PS molecule binding is not required, as the structure of the particular nanoscale material makes it possible to generate ROS. The characteristic examples of this group are fullerenes, titanium dioxide NPs, and zinc oxide (ZnO) NPs (see Figure 1B). The representatives of this second class of NPs have shown very good photostability and high photobleaching resistance [100], as well as a low toxicity, biological inertness, superior biocompatibility, and perfect photocatalytic activity [101,102,103,104]. However, insolubility in physiological conditions and aggregate formation are two common limitations observed in fullerenes and titanium dioxide NPs. Therefore, variable approaches have been analyzed, such as PEG-ylation [105,106], encapsulation [107,108,109,110,111], or the combination of fullerenes with aptamers. As an example, the highly specific targeting of epithelial growth factor receptor (EGFR) in human lung carcinoma A549 cells with trimalonic acid-modified C70 fullerenes (TF70) conjugated with aptamer R13 leads to a good binding capability and efficient cell killing after irradiation [112].

The ZnO NPs have been the most extensively studied nanomaterials from this class of NPs, due to their high biocompatibility and low toxicity, with the Food and Drug Administration (FDA) recognizing ZnO NPs as safe [113]. Specifically, they absorb radiation in the UV spectrum and are able to produce ROS after irradiation in aqueous suspension, which causes antibacterial and anticancer effects [114,115,116,117,118,119]. Moreover, Wang et al. (2017) proved the effective downregulation of CD44, the predicted cancer stem cell (CSC) surface marker, leading to sensitization to doxorubicin (DOX) treatment in human breast adenocarcinoma MDA-MB-231 and human ovarian serous carcinoma NCI/ADR-RES experimental cancer cell lines. Moreover, the inhibition of cancer cell adhesion and migration, and the prevention of a spheroid formation were observed [120]. Despite the great potential of ZnO NPs, some attributes must be improved for their successful clinical application, such as ZnO modification, for the purposes of long wavelength absorption and higher photostability [121].

### 2.3. The Third Class of NPs—Energy Transducers of PSs

The third class of NPs, represented by energy transducers of PSs is composed of NPs that not only fulfill the role of PS vehicles but can also actively participate in energy transfer to the attached PS. The utilization of these NPs has a great potential because it enables shorter and high-energy wavelengths to be applied, which are transferred by NPs to the associated PSs. The use of energy transducers represents a potential approach for cancer treatment in relation to PDT, as it also has a great ability to widen PDT application for deep-sited tumors. In this context, X-ray-activated NPs are an extensively studied method as they can absorb deeply penetrating X-ray radiation and emit luminescence in the visible spectrum, which subsequently leads to the activation of the attached PSs [122,123]. Some representatives of the third class of NPs, such as quantum dots, can generate ROS themselves, but the total ROS production is relatively low. Based on this, the higher therapeutic potential lies in their conjugation with PSs [124]; however, the spectral characteristics of many conventionally utilized PSs are not ideal for their combination with the third class of NPs, as it could be shown on e.g., two-photon-absorbing NPs for example. Therefore it is important not only to improve the characteristics of the NPs, but also to develop appropriate novel PS molecules [125]. To summarize, X-ray-activated NPs, quantum dots, two-photon-absorbing NPs, and up-conversion NPs could be included in this group (see Figure 1C) [126,127,128,129].

## 3. Problematic Attributes and Limitations of PDT

The era of potential cancer treatment using modern PDT started more than sixty years ago [130]. Since then, research in this field has been developing rapidly and is considered to be a very efficient modality for the treatment of various malignant and non-malignant diseases. The selective destruction of cancer cells with minimal toxicity towards non-cancer cells represents a significant advantage for their successful application in clinical use [131]; however, the improvement of the targeting characteristics of PDT is still crucial [132].

The therapeutic efficacy and success of PDT is based on three fundamental components—the properties of the PS, the spectral characteristics of light and its output power, and finally, the presence of molecular oxygen [133]. In comparison with conventional therapeutic approaches like chemotherapy or radiotherapy, PDT is not an invasive method, which consequently reduces the risk of infections and brings excellent cosmetic results [134].

PDT has also shown its applicability in the treatment of microbial [135] and viral infections [136,137,138]. Moreover, it is possible to use it to treat actinic keratosis, superficial, nodular basal cell carcinoma, Bowen´s disease, and some types of viral skin infections [134].

At the molecular level, the effect of PDT depends mainly on singlet oxygen [133]. The molecules of the PSs must be placed close to the targeted organelles at the time of irradiation as the half-life time of singlet oxygen (<0.04 µs) and the radius of its action (<0.02 nm) are short [139].

The sites that are most preferred for the accumulation of PSs are mitochondria, lysosomes, plasma and intracellular membranes, Golgi apparatus, and the endoplasmic reticulum. Controversially, accumulation in the cell nucleus is very rare [140]. Besides, the cell nucleus is not a preferred target of PDT because it can potentiate mutagenesis under certain conditions as a consequence of genetic material effects [55]. In general, intracellular damage of mitochondria and the endoplasmic reticulum is prevalently associated with apoptosis, whereas PDT targeted on lysosomes or the plasma membrane increases the possibility of necrosis [141]. Thus, the PSs that accumulate close to the mitochondria or endoplasmic reticulum have a higher application potential. It is clear that the allocation of PSs within the tissue and cells has a great impact on the outcome of PDT. Firstly, the distribution of PSs among organelles depends on the transport efficiency of the PS molecules into the intracellular environment. However, the previously mentioned aggregate formation of PS molecules significantly limits its uptake and reduces the efficiency of PDT [82,142]. Thus, the search for novel PS solvents represents one of the essential lines of investigation in PDT research [83]. Furthermore, the systemic administration of drugs leads to their unwanted interaction with the surrounding environment. Therefore, poor penetration is not a terminally limiting factor that restricts the clinical use of many PSs. These interactions could also decrease or even fully reduce a desired pharmaceutical effect [143], which has been observed in the case of neutral leuko-methylene blue molecules, where the cationic reduction of methylene blue molecules resulted from their systematic application [144,145,146].

The higher accumulation rate of PSs observed outside the neoplastic section of the tumor mass or even in healthy tissues and skin is associated with their damage after irradiation and could contribute to tumor development [132]. The distribution kinetics of PS molecules [147,148] or, more precisely, the molecular mechanisms affecting their influx and efflux cell characteristics are probably the fundamental factors modulating the status of PS accumulation in particular tissues or cells [149]. Furthermore, we assume that there is no strict border between the mentioned mechanisms, but that they are both interconnected.

Besides the accumulation of PSs, another important factor affecting the treatment efficacy or failure is the phenomenon of MDR. The concept of MDR is not novel, as it has been very extensively studied over the last few decades [10,150,151,152]. Initially, only the reduction of chemotherapy efficacy was attributed to MDR [10], but since the 1990s, there has been a growing body of evidence highlighting the fact that MDR exceeds the borders of chemotherapy and could affect other therapeutic approaches, even PDT. Currently, the mechanism of MDR is greatly associated with the overexpression of ABC transporters, and MDR-associated protein-1 (MRP1/ABCC1), breast-cancer-resistant protein (BCRP/ABCG2), and P-glycoprotein (P-gp/ABCB1) have been the most extensively studied representatives [5]. In physiological conditions, ABC membrane transporters fulfill an irreplaceable role in the transport of toxic molecules out of the intracellular space using the energy from ATP hydrolysis. This mechanism prevents the intracellular accumulation of toxic compounds and protects the cells from damage [153]. A higher expression of these efflux pumps has been observed for example in the intestine, blood–brain barrier, and blood–testis barrier [154]. ABC transporters have also been observed in other internal organs, such as the liver and kidney, where they take part in detoxification [155]. Their presence in the placenta [156] is associated with the protection of the fetus from toxic factors in the maternal circulation [157]. Interestingly, the significant expression of ABCG2 transporter has been observed in the cell membranes of hematopoietic progenitor cells and other stem cells where their presence is linked with the proliferation and maintenance of the stem cell phenotype. In cancer cells, the expression of ABCG2 is related to the presence of “side population” (SP) phenotype. The SP cells are resistant to certain chemotherapeutic drugs, thanks to their higher efflux activity. Moreover, the SP fraction actively supports tumor formation and its progression [158]. Due to the fact that ABCG2 is standardly expressed in stem cells, it has been suggested that it may also serve as one of the possible, but not universal [159], biomarkers of CSCs [160].

ABC transporters show a broad substrate specificity, including many therapeutic drugs and PSs, too. In 1994, Kessel et al. [12] identified copper benzochlorin iminium salt (CDS1) as a substrate of P-gp, and other PSs molecules have since been confirmed as substrates of P-gp, such as tetrabromorhodamine 123 [13], thiorhodamins, and selenorhodamins [14]. Additionally, protoporphyrin IX [16], hematoporphyrin IX [17], pheophorbide a [17], 2-[1-hexyloxyethyl]-2-devinyl pyropheophorbide-a [16,18], phytoporphyrin (phylloerythrin) [19], chlorin e6 [17], benzoporphyrin derivative monoacid ring A [17], hypericin [20,161], and iminoacridine [21,22] have been identified as substrates of ABCG2.

Besides the fact that many PSs are substrates of ABC transporters, they can also actively modulate the level of certain efflux pumps. Indeed, some recently published papers have detected an increased expression of BCRP in the lung cancer cell line A549 [159] or elevated BCRP and MRP1 levels after hypericin application in dark conditions in colorectal HT-29 [15,20], and ovarian A2780 and A2780cis cell lines [23]. Moreover, Jendželovská et al. [23] observed an enhanced MRP1 expression in A2780 and A2780cis cells only 6 h after treatment with 0.5 µM hypericin. In HT-29 cells, the elevated expression of MRP1 was observed even 16 h after the application of 0.1 µM hypericin concentration [15,20]. Jendželovský et al. (2019) stated that the elimination of hypericin from cancer cells represents one of the essential obstacles affecting the efficacy of PDT with hypericin (HY-PDT). The decreased intracellular level of PSs affected by BCRP were associated with a lower therapeutic efficacy of PDT, which was also observed in other PSs, such as protoporhyrin [17,18,162,163,164], chlorin e6 [17,165], pheophorbide [166], pyropheophorbide a [167], pyropheophorbide a methyl ester [17], pheophorbide a [168], 2-(1-hexyloxethyl)-2-devinil pyropheophorbide-a (HPPH, Photochlor) [18], benzoporphyrin derivative monoacid ring A (BPD-MA, Verteporfin) [18], aminolevulonic acid-protoporhyrin IX (ALA-PpIX) [169], and photofrin (PT) [170].

## 4. Nanoparticles as a Possible Solution for Reducing the MDR Effect in Cancer Treatment

As mentioned in the section above, the phenomenon of MDR represents a very serious, if not the most important, factor that significantly reduces the efficacy of PDT. However, the problem is even more complex because, as mentioned above, many PSs are not only the substrates of ABC transporters, but could even enhance the MDR effect via upregulating their expression. All things considered, the lower therapeutic effect of PDT is the consequence of cascade reactions, where the enhanced amount of ABC transporters limits the intracellular accumulation of PSs. The lower therapeutic efficacy accompanied by survival of the targeted cell fraction can result in tumor regrowth and higher malignancy, which was observed using in vivo experimental models [132,147,171,172,173]. Finally, the tumorigenic potential, which was characterized by the enhanced ability to repopulate the tumor, is a typical feature of CSCs [174]. Thus, novel medical approaches focusing on the reduction of the MDR mechanism could make significant progress in cancer treatment.

With this in mind, biodegradable natural or synthetic NPs carrying PSs were initially utilized for PDT, with polyester- and polyacrylamide-based NPs; liposomal NPs belong to the most extensively studied representatives of this category.

The analyses with tetanus toxoid prepared in liposomes clearly showed a greater antibody response in comparison with free toxoid. Moreover, after the repeated application of free toxoid, the experimental animals died. In contrast, the animals who were treated with toxoid prepared in liposomes preserved good health [78]. Later, multiple liposome modifications were analyzed to improve the membrane stability [175] and entrapment potential for a wide range of molecules like chemotherapeutics [176], PSs [177,178,179,180,181,182], or mRNA [183,184]. Interestingly, thanks to long-term research, alongside the COVID-19 pandemic situation, liposomes have been used as transporters in officially approved mRNA vaccines [185].

The data have shown that the utilization of biodegradable NPs could significantly improve solubility, the effectivity of PSs delivery [177,178,179], tumoricidal activity [178], wavelength absorption parameters of PSs, the PS accumulation ratio between the skin and the tumor, and the tumor regression potential [180], as well as their long-storing capability [181]. Moreover, Lima et al. (2013) showed that the utilization of lipid NPs with a core, stabilized by the surfactant known as solid lipid NPs (SLNs), significantly reduces the essential deficiencies of the conventional lipid NPs linked with the low entrapment efficiency of the PSs. Importantly, the structural modification did not induce the toxic or phototoxic effect in vitro. In relation to SLNs, the entrapment efficiency of hypericin was more than 80% higher [182,186]. In addition, using HEp-2 human larynx carcinoma cells, B16-F10 mouse melanoma cells [182], and Hep G2 human hepatocellular carcinoma cells [186], a higher absorption effectivity, higher photostability, lower photodegradation [182,186], more effective singlet oxygen production, and about 30% higher hypericin intracellular accumulation and 26% higher phototoxicity (in comparison to the experimental group treated with free hypericin) were detected. Thus, SLNs might help to partially overcome the enhanced efflux of PSs by transporter proteins, which is the typical manifestation of MDR, by increasing the intracellular PS content [182].

On the contrary, there are several pieces of evidence pointing to the fact that the higher PS encapsulation efficiency observed in SLNs [182,186,187] or polyactic acid polymeric NPs (PLA) has a negative effect on their photoactivity. Surprisingly, Zeisser-Labouebe (2006) observed a lowered photocytotoxic effect of encapsulated hypericin when compared to free hypericin on NuTu-19 cells, depending on the increasing encapsulation efficacy of PLA. The influence of drug loading on the phototoxic effect of biodegradable NPs could be explained by multiple parameters. The most likely explanation lies in the NP size, where particles with a diameter higher than 200 nm could significantly lower PDT effectivity as a consequence of their decreased permeability, and thus limit access to the tumor [186,188]. Another potential reason could be that PSs loaded into NPs with a smaller diameter may be closer to the surface of the NPs, and a more rapid release is therefore possible [187]. Observations where a higher drug loading capacity is paradoxically associated with the limited drug release capability of NPs are not only noted in relation to PSs. Mu and Feng (2003) observed a similar trend with the utilization of paclitaxel-loaded poly(DL-lactide-co-glycolide) (PLGA) NPs with a diameter about 400 nm, and Görner et al. (1999) clearly showed that larger NPs exhibit a slower release [189,190]. Using lidocaine loaded in poly(d,l-lactic acid) NPs varying in particle size from about 250 to 820 nm, they also suggested that the release profile of NPs is affected by a combination of the size and drug loading parameters of the NPs. The authors also suggested the creation of a heterogenous matrix with a higher drug loading in the NPs whose presence limits drug release. Therefore, the loaded drug must firstly be dissolved in these highly loaded NPs, which causes its slower release. In relation to PSs, the use of larger NPs (>200 nm) [186] could be associated with a higher rate of aggregate formation in these NPs, which could significantly restrict the photocytotoxic effect of PDT [187].

Naturally, biodegradable NPs are designed to load, deliver, and release particular molecules. Therefore the major drawbacks of biodegradable delivery systems are associated with the risk of PS efflux by the MDR mechanisms [143], and also with the persistence of the post-treatment accumulation of drugs in the skin and eyes, resulting in long-term phototoxic side effects [191].

### 4.1. Dynamic Nanoplatform (DNP)—The Concept of ^1^O_2_ Release to Target Cells Rather than the PS Itself

The concept of a dynamic nanoplatform (DNP) has been developed as a potential solution to the disadvantages presented [25,192]. The concept of the DNP enables the utilization of PDT methods that fundamentally differ from conventional approaches. The mechanism of the DNP is based on the encapsulation of PS into the porous NPs. Subsequently, the NPs are delivered to the tumor mass and are accumulated close to the cell membranes. During this phase of treatment, the applied light induces the generation of ROS. The structure of highly-specialized non-biodegradable NPs makes the release of ^1^O_2_ possible, subsequently preventing the diffusion of the PS from the carrier. The crucial factor for successful ^1^O_2_ delivery is based on the particle pore size, which must be smaller than the PS molecule, but larger than the O_2_ and ^1^O_2_ molecules, in order to enable them to pass through the particle shell. Subsequently, the generated ^1^O_2_ is able to target and damage the cell membranes [143]. The permanent encapsulation of PS molecules is crucial because the prevention of the direct interaction between PSs and ABC transporters limits the MDR effect in cancer cells [25,192].

In the last fifteen years, significant development and progress in the concept of the DNP have been observed. Currently the concept has shifted to multifunctional treatment approaches, which will be discussed below. The use of nanocarriers permanently retaining particular drugs is not suitable from the point of view of conventionally applied treatment methods, and the application potential is strictly limited to the field of PDT [30].

**Figure 2:**
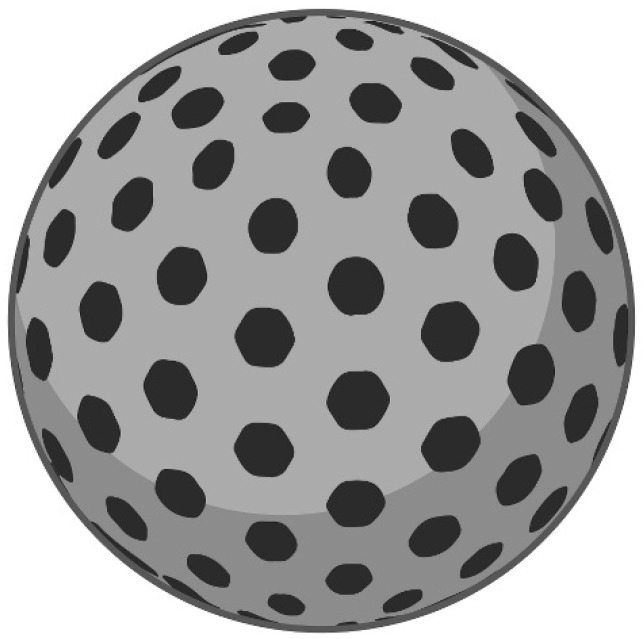
Mesoporous silica nanoparticle as a typical representative of DNP concept.

Despite the fact that the theoretical basis of the DNP concept has clear contours, its practical use is strictly limited by the properties of the NPs. Apart from the generally preferred attributes of NPs such as their biocompatibility, a spherical shape, and a uniform size with diameter under 200 nm [24,186], we must emphasize that light transparency, photochemical inertness [32], monodispersity [31], a porous skeleton structure, higher thermal resistance, and stability preservation in extreme pH [30] are fundamental requirements for materials when dealing with a DNP. The most promising materials, fulfilling the requirements of DNP concept, seem to be polyacrylamide [143] and polyacrylamide combined with polyethylene glycol (PEG) [25], and organically modified silicate and silica NPs (see Figure 2) [143] with different modifications like phosphonate [36], polyethyleminine and PEG [27], which served as a coating layer for its functionalization [35,43,47].

**Figure 3:**
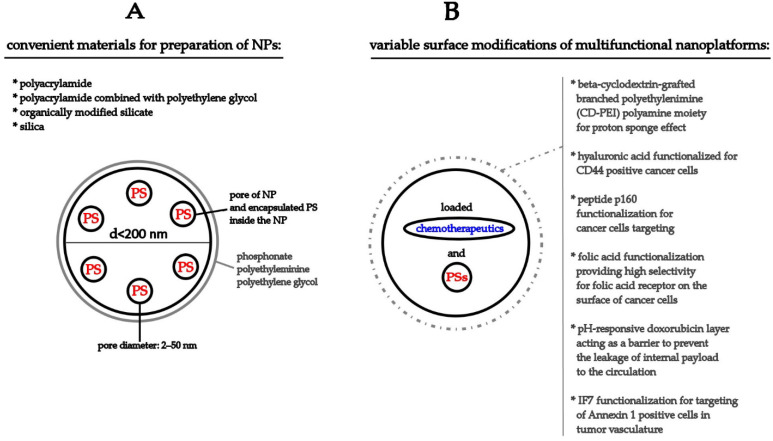
Graphical representation of NPs convenient for application in DNP concept of PDT (**A**) and multifunctional therapeutic approaches for application in combined chemo-PDT (**B**).

As will be discussed below, silica NPs have been associated with most widely used carriers for the research of PDT in connection with DNPs. On account of this purposes, porous NPs that were generally marked as mesoporous silica NPs (MSNs), with a particle size of 30–200 nm, a large surface area (up to 1000 m^2^/g), and a pore volume (>0.9 cm^3^/g) with a typical pore dimeter of about 2–50 nm were utilized (see Table 1 and Figure 2). There is also some evidence of the application of hollow-type mesoporous silica NPs (HMSNPs) (types: MCM-41 or SBA-15). HMSNPs are structurally similar to the MSNs but are characterized by their hollow core–mesoporous shell structure and enhanced loading capacity (>1 g drug/1 g silica) [193]. For a better understanding of the presented article, we are going to work with two terms only—MSNs and HMSNs. The original definition of NPs used by the authors will be stated (see Table 1 and Table 2, Figure 2 and Figure 3A).

In traditional PDT, the irradiation can induce necrotic or apoptotic cell death [140,194,195]. As mentioned above, the balance between apoptosis and necrosis depends on the intracellular location of the PSs and subsequently on the related damage of the particular cell organelles [133]. Moreover, other features such as the dose of irradiation and the cell type represent additional crucial factors affecting the type of cell death. In connection with the DNP in its original form, ^1^O_2_ delivery to adjacent cell membranes could preferentially induce necrosis [25,143].

Roy et al. [24], as pioneers, observed an active uptake of photoactive water-insoluble PS 2-devinyl-2-(1-hexyloxyethyl) pyropheophorbide encapsulated in silica NPs into the cytosol with the use of cervical carcinoma HeLa and ovarian adenocarcinoma UCI-107 cell lines. Interestingly, the authors observed that an entrapped drug is more fluorescent in aqueous medium than a free drug, being able to produce ^1^O_2_ more effectively. Similar results were also observed when 2-devinyl-2-(1-hexyloxyethyl) pyropheophorbide was used as the PS [35].

Zhu et al. (2011) prepared MSNs with a very small diameter (about 37 nm) using the hydrothermal treatment and PEG [194]. The fluorescence intensity of silicon phthalocyanine dichloride (SiPcCl_2_) in HeLa cells was significantly higher in comparison to the free drug. In addition, the PS was detected not only in the cytoplasm, but also in a cell nucleus. Interestingly, the phenomenon of intranuclear transport cannot be observed in the case of pure SiPcCl_2_. The authors also proclaimed that MSNs have the potential to double the efficacy of ^1^O_2_ production and could facilitate a photo-oxidation reaction [26]. These observations could be associated with an approximately 6.3 to 7.0-fold higher photocytotoxic effect of encapsulated SiPcCl_2_ in comparison to its free solution. In contrast to biodegradable NPs [186,188], there was a promising antitumor effect, which was accompanied by an excellent loading capacity of about 82.6% with encapsulated SiPcCl_2_ [26]. Based on the previous context, the complete MSN boosts the phototoxic effect of the PS [26,32], enhances the quantum yield of encapsulated PS [29], and fulfills the role of a nanoreactor in the PDT reaction [26]. The enhanced photoactivity of encapsulated PSs could be prevalently associated with the rigorous protection against its aggregation [32]. Ross et al. (2004) even proclaimed that NPs with encapsulated PS permit ROS generation, and they could be regarded as an individual photosensitizer.

Leaky vasculature and poor lymphatic drainage are typical characteristics of the microenvironment of solid tumors, which subsequently enable the permeation of NPs from the blood vessels into the tumor, where they are retained. This phenomenon is termed as the enhanced permeability and retention effect (EPR) [195]. Results of in vivo analyses showed that structurally modified MSNs are prevalently accumulated in tumors as the consequence of EPR [33,70,196,197]. Moreover, a relatively low accumulation was also detected in the liver and spleen [27]. If non-modified MSNs were utilized, the prevalent accumulation was detected in the liver of the experimental animals [34]. Modified MSNs have also emerged as promising carriers for PDT, thanks to their chemical inertness, large surface, and easily modified volume, pore size, and surface area [198]. Additionally, data shows that the biocompatibility and the level of encapsulated PS could be improved with the utilization of PEG. The functionalization of MSNs with polyethylenimine enhances their intracellular accumulation as a consequence of the “proton sponge” effect, which is associated with the endolysosomal escape of MSNs [27].

The prevention of water and PS interactions is considered to be a significant benefit of silica NPs [31] and is associated with the long term persistence of the photoactive conditions [28,29,32]. This can be proven using the findings of He et al. (2009), who utilized highly hydrophobic methylene blue (MB) encapsulated in phosphonate coated MSNs. After 10 days of incubation in water or PBS, 95% of the fluorescence was preserved, and 90% of the fluorescence intensity was detected when analyses were performed in a serum. Moreover, the intensity of the emission signal generated from the encapsulated MB was almost nine time higher than free PS. Similar results were detected in human cervical adenocarcinoma HeLa cells using SiPcCl_2_ [26].

Interestingly, the encapsulation of phthalocyanine and subsequent light induction significantly inhibited the growth of hepatocellular carcinoma derived from H22 cells, which led to the prolonged survival of experimental animals [27].

In 2015, Yang et al. designed highly sophisticated MSNs, represented by a three-in-one system. The inner cavity served as a reservoir to encapsulate a chemotherapeutic agent, while the C_60_ molecules acted as a PS for PDT and also as a fluorescent agent for imaging. The authors indicated a pH-induced release of DOX from the NPs and a remarkable therapeutic efficacy at the same time, thus requiring a lower drug dosage. This was the first presented report of multifunctional chemotherapy, PDT, and a fluorescent imaging nanosystem using MSNs. One month before the mentioned article was published, Spring et al. (2015) noted that “it is conceivable that resistance induced by one treatment might be overcome by another treatment” [199].

### 4.2. Multifunctional Therapeutic Nanoplatforms as a Highly Effective Novel Treatment Modality to Reduce the MDR Effect in Tumors

Over approximately the last five years, the development of multifunctional PDT and chemotherapy nanosystems has represented one of the essential motifs in the field of MDR research. As noted, chemotherapy is one of the most important therapeutical modalities for cancer treatment, with treatment regimens containing platinum drugs being administered in about half of cancer patients [196]. Cisplatin was the first platinum-based anticancer drug and has been proven as one of the most efficacious drugs to treat cancer since 1978, when it was approved for clinical use [197,200]. However, the efficacy of cisplatin is hindered by the inherent or acquired resistance of cancer cells [201]. Data indicates that the lowered efficacy of cisplatin is affected by multiple factors [202,203], but its lower intracellular accumulation seems to be the primary cause of its resistance. A human copper transporter protein 1 (hCTR1) is the major influx transporter of cisplatin; in cisplatin-resistant cancer cells, its downregulation was detected [204,205,206]. The results of some analyses have shown that the use of a nanosystem represents a promising strategy for circumventing cisplatin resistance [207]. Zhang et al. (2016) constructed MSNs for a cisplatin prodrug and chlorin e6 (Ce6) co-delivery, to enable combined chemo-PDT against cisplatin resistant cancer cells A549R. The intracellular cisplatin accumulation in A549R cells with Ce6/cisplatin MSNs was about nine time higher than cisplatin alone, thanks to the endocytosis transport mechanism. Subsequently, MSNs/Ce6/cisplatin prodrug NPs were released to the cytoplasm due to the beta-cyclodextrin-grafted branched polyethylenimine (CD-PEI) polyamine moiety on the surface of NPs, which exhibited a strong proton sponge effect and facilitated the rupture of the lysosome. This was accompanied by about 20-times higher cytotoxicity of the MSNs/Ce6/cisplatin prodrug in contrast to the free cisplatin. This effect was further enhanced by light activation, which confirmed the synergistic effect of the individual components of the MSNs/Ce6/Pt NPs when compared to free cisplatin. Similar to cisplatin, DOX is another chemotherapeutical drug with a strong antitumor effect on a wide spectrum of tumors, such as brain and prostate cancer, and it is obviously nowadays considered to be the most effective chemotherapeutic drug to treat breast cancer [208]. However, DOX, and especially its repeated administration, can induce MDR mechanisms in cancer cells, as well as life-threatening cytotoxicity. Therefore, targeted drug delivery systems or combined treatment approaches have been developed [53,209,210,211].

Multiple approaches in relation to the development of PDT and chemotherapy combination strategies using MSNs have recently been analyzed. To minimize the side effects and enhance PS delivery, pH-sensitive [51,52] or hyaluronic acid (HAC)-functionalized MSNs have been prepared [54].

The results of in vitro analyses realized on human breast cancer cells MCF-7 [52] and MCF-7/ADR cells [51] showed some interesting findings. In both cell lines, the synergistic effect of photoactivated rose bengal [52] or Ce6 [51] and chemotherapy was observed. Moreover, the excellent loading properties of Ce6 [51], indocyanine green [54], and DOX were observed in magnetic MSNs [51] or MSNs [52]. In addition, a cumulative DOX release was observed in both cases despite the fact that the pH-sensitive mechanism was completely different. In the case of magnetic MSNs, the DOX-releasing mechanism was regulated by poly(asparaginyl-chidamide) [51] while Yan et al. (2018) designed MSNs with DOX linked to the shell of the NPs by a hydrazone bond. This method of NP preparation makes it possible to create a pH-responsive DOX layer acting as a barrier to prevent the leakage of internal payloads in the circulation [52].

In contrast to the above-mentioned findings, there is evidence of the low drug efficacy of MSNs not enhancing the cytotoxic effect of MSNs against cancer cells [56]. Therefore, HMSNs with large cavities could solve the problems of lower drug loading that is observed in MSNs. The excellent loading of DOX and indocyanine green in double pH/CD44-functionalized HMSNs (marked as: ID@HMSNs-B-HA) coated with dopamine-modified HAC has been recently presented. Similar to MSNs [33,34,35], excellent biosafety in the dark and enhanced cytotoxicity of ID@HMSNs-B-HA was observed in HeLa cells in contrast to the experimental groups treated with non-functionalized NPs, or DOX and rose bengal alone [54].

There is no doubt that in the context of MDR, silica NPs represent one of the most studied and promising materials. Moreover, their unique properties make it possible to design highly specialized, biocompatible multifunctional hybrid nanoplatforms to significantly reduce MDR in cancer cells. Nevertheless, there is evidence pointing to the fact that their lower drug loading could limit their utilization in some cases [56]. Meanwhile there is proof of an excellent loading capacity being achieved [26] or a synergistic anticancer effect, in spite of the relatively low loading of PS [53]. There could be multiple reasons affecting the drug loading parameters of the nanosystem that are related to their structural modifications and to the characteristics of the encapsulated drugs too. Additionally, there is multiple evidence that HMSNs represent one of the possible solutions for enhancing the drug loading efficiency of silica NPs [41,54].

In relation to this, the potential of other nanoplatforms was also analyzed [43,44,45,46,49,57] and some novel nanoplatforms were even developed [46,47].

The importance and relevance of novel analyzed nanoplatforms is supported by the fact that even a direct modulatory effect on ABC transporters was observed.

The novel cerium oxide NPs (nanoceria) with Ce6, conjugated in combination with free DOX [46] or with polymeric mixed micelles with loaded mitoxantrone [48] decreased the protein expression (detected by Western blot) [46] or the activity of P-gp (detected by a multidrug resistance assay kit), and they also reversed MDR [48] in drug-resistant human breast MCF-7/ADR cancer cells [46,48]. As mentioned above, the application of inorganic ceria nanocomposites functionalized with folic acid (FA) increased the cellular uptake of Ce6 into the lysosomes, which led to ROS production after PDT. Moreover, the total effect of combined Ce6 and DOX therapy was higher than the single treatment in MCF-7/ADR-resistant tumor cells. Apart from the significant tumor-targeting abilities, tumor growth inhibition was also detected [46]. Furthermore, Baglo et al. (2019) showed that a benzoporphyrin derivative (BPD) is no longer a substrate for ABCG2 and became a weaker substrate for P-gp when porphyrin-lipid nanovesicles (LysoPC-BPD) were used as a transport vehicle on the model of human breast P-gp-overexpressing MCF-7 TX-400 and ABCG2-overexpressing MCF-7 MX100 cancer cells. Moreover, the intracellular level of LysoPC-BPD was maintained for up to 16 h in MCF-7 TX400 cells. This finding correlates with the observations of other authors that used pH-sensitive NPs [43] or NPs functionalized with a cell-specific marker, Annexin 1 [49] or CD44 [44]. Interestingly, similarly to silica NPs [33,51], poly (ADP-ribose) polymerase (PARP) cleavage [48] and cell death (apoptosis, autophagy, and oncosis) was detected after the treatment [46,48].With the utilization of a polymeric prodrug (PMP) encapsulated with a near infrared fluorophore (DEB-BDTO) as the PS, along with tariquidar (TQR) as an MDR inhibitor, the synergistic effect of PDT and chemotherapy was observed in SKOV-3 and SKOV-3/MDR cells. In addition, these DEB/TQR@PMP micelles inhibited tumor growth in tumor-bearing mice in a stronger manner than PDT or chemotherapy alone (see Figure 3B) [47].

Recent findings have also shown, that combined therapy could represent a very promising therapeutic approach in CSC treatment. The logical approach uses conjugates consisting of a specific antibody serving as guidance towards the CSC population, together with a carrier for the PS and chemotherapeutic agent. This approach to eliminating CSCs, while increasing overall PDT efficiency, was introduced by Yang et al. [61] in 2019 on the model of liver CSCs derived from CD133-positive and 3D-propagated Huh7 and CCLP-1 cells. They used organoplatinum (II) metallacaged-based NPs combining cis-(PEt_3_)_2_Pt(OTf)2 with 5,10,15,20-tetra(4-pyridyl) porphyrin (TPP) and disodium terephthalate (DSTP), using amphiphilic micelles with RGD-PEG-*b*-PEBT to enable a higher stability and possible longer circulation in the blood stream, together with selective tumor accumulation via the binding ability of Cyclo(Arg-Gly-Asp-D-Phe-Lys) to the α_V_β_3_ integrin receptor on the surface of the cancer cells. A synergistic effect was observed in liver CSCs, which was characterized by a decreased mitochondrial membrane potential and a dramatic increase in the level of apoptosis-related proteins and apoptosis itself. Moreover, other features of CSCs were suppressed, such as the migration and spheroid-forming abilities. Additionally, combined photochemotherapy successfully diminished CSCs, inhibited their migration and clonogenicity, and most significantly decreased their tumorigenic potential. Interestingly, combined photochemotherapy was also effective in the ablation of CSCs that were located in the core of the spheroids. In addition, the supramolecular cage structure protects PS aggregation that could potentiate ROS generation.

Considering lung carcinomas, different CSC markers have been identified so far; however, CD133 has a great significance in the presence of the CSC phenotype [212].

Therefore, targeting CD133-positive lung cancer cells could represent a possible solution for the eradication of CSCs in the treatment of lung cancer. Application of gold CD133-functionalized NPs with phthalocyanine chloride tetra sulfonic acid (AlPcS_4_Cl) bound to the surface significantly enhanced its intracellular accumulation in CD133-positive A549 lung carcinoma cells and was accompanied by an enhanced efficiency of PDT, as demonstrated by morphological changes, decreased viability, increased cytotoxicity, and the enhancement of early apoptosis. Despite the higher accumulation of PS in complex bioconjugates, the differences between groups treated with PDT using AlPcS4Cl-AuNP with or without antibody were observed only at the level of viability. Other parameters indicating PDT efficacy were similar. Moreover, the PS accumulated in the cytoplasm if functionalized NPs were applied, while its accumulation was also detected in the cell nucleus in the experimental groups treated with free PS, which could negatively affect the genetic material of targeted cells after photoactivation [59].

Very similar and perspective results were observed using c60 fullerene-silica nanoparticle systems that were surface-decorated with HAC, in order to target the variant CD44, overexpressed in breast cancer cells (NPs marked as HC60S-DI) [60]. The uniqueness of these NPs is based on the interconnection of three therapeutic approaches: PDT, chemotherapy, and photothermal therapy. Focusing on PDT and chemotherapy, the authors noted the excellent encapsulation efficiency of DOX and indocyanine green (>90%), which consequently did not limit the phototherapeutic and photocytotoxic properties of the NPs. The highest ROS generation was observed in the MDA-MB-231 cells treated with HC60S-DI; in contrast, no fluorescence was detected if free DOX and indocyanine green was applied to MDA-MB-231 and MCF-7 cells. The surface-decorated with HAC provided a double function—the targeting of CD44 overexpressed on breast CSCs and the prevention of aggregate formation of NPs in aqueous solution. Moreover, after the application of HC60S-DI with high drug loading, complete tumor destruction was observed in three out of five mice; the remaining two tumors were smaller, with extensive apoptosis and necrosis declared, in comparison to other experimental groups.

Since hypoxia is a typical trait of the solid tumor microenvironment, could also represent a supportive niche for CSCs and is an obvious obstacle in PDT effectivity, we find it beneficial to seek therapeutic modalities with preserved efficacy under such low oxygenation. The application of a type II reaction is significantly limited because it involves energy transfer to oxygen molecules; hence, the presence of oxygen molecules is critical in the surrounding environment. In 2014 Usacheva et al. [58] analyzed the possibility of a less dependent, type I reaction application against MCF-7, 4T11, SKBR3, and MDA-MB-231 breast cancer cells using polymer–surfactant NPs (composed of sodium alginate and docusate sodium with encapsulated MB) to enhance ROS formation under hypoxic conditions. As expected, a higher cytotoxic effect was observed in normoxic and hypoxic conditions when compared to free MB. Moreover, a reduced colony formation, decreased primary and secondary mammospheres’ formation, and diminished ALDH^+^ fraction regardless of oxygenation were observed in the experimental groups treated with NP-encapsulated PS.

To sum up, excellent progress in the field of MDR research has been observed over the last ten years. Interestingly, all data have shown that the combination of PDT and chemotherapy dramatically improved cytotoxicity in drug-resistant tumor cells with the utilization of NPs, whereas the separate administration of these therapeutical approaches could induce multiple side effects and MDR in cancer cells [19,22,23,132,161].

On the basis of these novel findings, the above-mentioned statement by Spring (2015) keeps its validity. Moreover, we could suggest that the parallel combination of two selectively tumor-inducing resistance mechanisms using progressive nanocompounds in the role of nanocarriers might overcome MDR in cancer.

However, the majority of information is obtained from in vitro analyses (see Table 1 and Table 2) and only limited spectra of PSs and chemotherapeutics were analyzed from combined therapy. Therefore, the research of other promising molecules utilizing NPs in the context of MDR is crucial for PDT and chemotherapeutic protocol improvement.

## Figures and Tables

**Table 1:** Application of silica NPs for PDT in the context of DNP.

In Vitro/In Vivo Model	PS/Chemotherapeutic Agent	PS Administration	NS or Solvent	Irradiation Conditions	Observed Effects	References
ovarian serous carcinoma (UCI 107), human cervical adenocarcinoma (HeLa)	2-devinyl-2-(1-hexyloxyethyl) pyropheophorbide		silica NPs	650 nm; 1.4 mW/cm^2^; 10 min	* average size of NPs = 30 nm * NPs were accumulated in cytoplasm *↓ toxicity in dark conditions * significant increase in cell death was observed, if NPs with PS were applied	[24]
human breast adenocarcinoma (MCF-7), melanoma (MDA 435); rat experimental model	PT	i.v.	* RGD peptide modified PAA NPs coated with PEG (RGD peptide modified polyacrylamide (PAA) core with a surface consisting of PEG)	630 ± 3 nm; 700 mW; 3 min	* average size of NPs: 40 nm * massive necrosis after PDT was observed, if NPs with bound PS were applied * NPs had a potential to selectively bind to αvβ3 integrin on the surface of cancer cells * no toxicity was detected in experimental animals four weeks after NPs application	[25]
human cervical adenocarcinoma (HeLa)	HA		silica NPs	-------	* average size of NPs = 130 nm * micro-hole size (<0.53 nm) makes possible to release only the ^1^O_2_ from the NPs, molecules of PS were retained *↑ quantum yield of encapsulated HA * HA detected in HeLa cells * in dark condition no toxicity of encapsulated HA was detected *↑ effectivity of PDT with encapsulated HA	[29]
human cervical adenocarcinoma (HeLa)	HA		silica NPs	0–25 J/cm^2^	* average size of NPs: 110 nm *↑ fluorescence intensity of encapsulated HA *↑ photostability and ^1^O_2_ generation of encapsulated HA * active uptake of HA if NPs were used * low dark toxicity of encapsulated HA * apoptosis was observed and ↑ photodamage after PDT with NPs utilization	[30]
human cervical adenocarcinoma (HeLa)	PpIX		silica NPs	532 nm, 2 mW/cm^2^; 2 min	* average size of NPs: 25 nm * encapsulated PpIX emitted ↑ fluorescence than free PpIX * encapsulated PpIX had net cationic charge and the HeLa cells had anionic charges on their membranes. This interaction facilitated the uptake of the cationic amino-functionalized NPs by the HeLa cells * HeLa cells were successfully destroyed 8 min after PDT with encapsulated PpIX * after PDT necrosis was detected	[31]
esophageal squamous cell carcinoma (KYSE 510)	mTHPC		silica NPs	600–700 nm; 2 mW cm^−2^; 0.12 J cm^−2^	* average size of NPs: 24–47 nm * spherically shaped NPs * the molecules of mTHPC were included inside the NP in monomeric form * complete loss of viability after PDT in cells treated with 1.25 uM of encapsulated mTHPC was detected * encapsulation did not affect intracellular fluorescence distribution of PS * mTHPC was largely localized in GA and ER * free mTHPC was taken up by the cells more efficiently than mTHPC in NPs but the cytotoxic effect was equal	[32]
melanoma (A375; B16-F10)	Pc4		silica NPs	600–700 nm; 6.6 J cm^−2^; 15 min	* average size of NPs: 25–30 nm *↑ fluorescence lifetime, photostability and reduced photobleaching rate of encapsulated Pc4 * no dark toxicity of encapsulated and free Pc4 *↑ phototoxicity and apoptosis rate detected, if Pc4 was encapsulated in comparison to free solution *↑ Pc4 was localized in mitochondria, if NPs were utilized	[33]
human colon cancer (HCT 116, HT-29), human breast adenocarcinoma (MCF7, MDA-MB-231), human skin carcinoma (A431), LLBC37, human lung carcinoma (A549); female athymic Swiss nude mice	PpIX	i.v.	silica NPs	630 nm; 4 mW cm^−2^	* average size of NPs: 10–60 nm * in all tumor types, encapsulated PpIX was more efficient than free PpIX *↑ ROS production in HCT 116 and HT-29 cells, if encapsulated PPIX was used * tumor models reached maximal accumulation of NPs at different time points: 2 h for glioblastoma, 16 h for A549 and 20 h for HCT 116 *↑ NPs accumulation was detected in the liver than in the tumor	[34]
human cervical adenocarcinoma (HeLa)	HPPH		silica NPs	850 nm	* average size of NPs: ≤30 nm * encapsulated HPPH produced ^1^O_2_ in water * active uptake of encapsulated PS by tumor cells was observed and fluorescence of NPs in cytoplasm was detected * cell necrosis after PDT with encapsulated HPPH was detected	[35]
human cervical adenocarcinoma (HeLa); male athymic BALB/c nude mice	MB	i.v.	phosphonate coated silica NPs	in vitro: 635 nm; 27.5 mW/cm^2^; 0–45 min, in vivo: 635 nm; 500 mW/cm^2^; 5 min	* average size of NPs: 105 ± 6.8 nm * phosphonate coated NPs with encapsulated MB have 8.6 time ↑ emission signal than non phosphonated NPs * encapsulation of MB effectively prolonged the fluorescence properties of MB in water and serum * >80% cytotoxicity of encapsulated MB in vitro even in the lower concentration (0.7 mg/mL) * in vivo: after PDT with encapsulated MB necrosis of tumors was detected	[36]
human colon adenocarcinoma (SW480)	PHPP		silica coated magnetic NPs	488 nm; 4.35 J/cm^2^	* average size of NPs: 20–30 nm * concentration dependent cytotoxicity of encapsulated PHPP * significant ^1^O_2_ generation with NPs utilization was observed * no dark toxicity	[28]
human cervical adenocarcinoma (HeLa)	SiPcCl_2_		MSNs	600–710 nm, 0.8 mW cm^−2^	* average size of NPs: 37 nm * encapsulated PS can double the efficiency of ^1^O_2_ generation * in the dark almost no cytotoxicity of encapsulated PS was detected * 6.3 to 7.0 fold ↑ photocytotoxic effect and fluorescence intensity of encapsulated PS in comparison to free PS * encapsulated PS was detected not only in cytoplasm, but also in cell nucleus *↑ loading capacity of PS (82.6%)	[26]
murine hepatocellular carcinoma (H22); female BALB/c nude mice	phthalocyanine	i.v.	polyethylenimine and polyethyleneglycol functionalized MSNs (PEG-PEI-MSNs/ZnPc)	in vitro: 680 nm; 3–36 J/cm^2^; 10 mW/cm^2^; 5–60 min, in vivo: 680 nm; 12 J/cm^2^; 200 min	* average size of NPs: 50 nm and pore: 3.3 nm * encapsulated PS effectively produces ^1^O_2_ * functionalized NPs have a high efficiency to escape from the lysosome into the cytosol *↑ cell death in PEG-PEi-MSNs/ZnPc was in comparison to other experimental groups * PEG-PEI-MSNs/ZnPc, could produce ↑ (>80%) phototoxicity with a final concentration of ZnPC at ≥0.26 µM * NPs with ZnPc were prevalently accumulated in tumor * PEGylation of MSNs ↑ accumulation in comparison to non-PEGylated NPs * tumor growth was significantly suppressed in the PEG-PEI-MSNs/ZnPC-PDT experimental group	[27]

*, particular information related to MDR; ↑, means increase in observed parameter; ↓, means decrease in observed parameter; -------, the parameter was not provided by the authors; HA, hypocrellin A; HPPH, 2-[1-hexyloxyethyl]-2-devinyl pyropheophorbide-a; i.v., intravenous; MB, methylene blue; MSNs, mesoporous silica nanoparticles; mTHPC, meso-tetra-hydroxyphenyl-chlorin; NPs, nanoparticles; NS, nanosystem; Pc4, silicon phthalocyanine; PHPP, 2,7,12,18-tetra-methyl-3,8-di-(1-propoxyethyl)-13,17-bis-(3-hydroxypropyl) porphyrin; PpIX, protoporphyrin IX; PT, photofrin; SiPcCl_2_, silicon phthalocyanine dichloride.

**Table 2:** Application of combined chemo-PDT with NPs and functionalized NPs for the purposes of MDR effect reduction.

In Vitro/In Vivo Model	PS/Chemotherapeutic Agent	PS Administration	NS or Solvent	Irradiation Conditions	Observed Effects	References
human breast adenocarcinoma (MCF-7)	fullerene (C_60_)/DOX		mesoporous hollow silica-fullerene NPs (MHSF); silica-fullerene NPs (SSF)	UV irradiation; 5 min	* average size of NPs: 50 ± 7 nm *↑ generation of ^1^O_2_ by MHSF in comparison to SSF due to enhanced porosity in the silica framework of MHSF * 10× ↑ loading capacity of MHSF in comparison to SSF *↑ DOX release rate from MHSF in acidic environment than in neutral environment * excellent biocompatibility of MHSF * PDT with MHSF induced ↑ cell inhibition in comparison to SSF * silica framework effectively minimizes ^1^O_2_ quenching	[41]
human breast adenocarcinoma (MCF-7), human embryo skin fibroblast (ESF)	HB		p160-MSN-HB	480 nm; 10 min	* functionalization of MSN on p160 leads to significantly ↑ accumulation in MCF-7 cells in comparison to non-functionalized MSNs * significant ↓ cell viability in p160-MSNs-HB-treated group in both cell lines in comparison to other experimental groups	[42]
murine melanoma (B16-F10)	5ALA		hollow MSNs functionalized by folic acid	635 nm; 25 mW cm^−2^; 15 min	* average size of NPs: 150 nm * functionalization of NPs with folic acid leads to NPs internalization by endosomal route	[50]
human breast adenocarcinoma (MCF-7), DOX resistant human breast adenocarcinoma (MCF-7/ADR); BALB/c nude mice	Ce6/DOX	i.v.	magnetic mesoporous silica-based nanocomposite (MMSN)	in vitro: 660 nm; 3 min, in vivo: 660 nm; 10 J/cm^2^; 5 min	* average size of NPs: 135–145 nm *↑ production of ^1^O_2_ in free Ce6 NPs * MMSNs produce sufficient level of ^1^O_2_ * significantly ↑ releasing of DOX from NPs in acidic pH *↑ intracellular uptake of encapsulated DOX in comparison to free DOX *↑ cytotoxicity of photoactivated MMSNs in MCF-7/ADR cells in comparison to free DOX * no dark toxicity of MMSN *↓ migration and invasion and apoptosis detected after irradiation with MMSN in MCF-7/ADR cells * good biocompatibility of MMSNs	[51]
human breast adenocarcinoma cancer (MCF-7)	ICG/DOX		pH-sensitive MSNs	532 nm; 0.5 W/cm^2^; 10 min	* average size of NPs: 218 nm * in pH 5.5 cumulative release of DOX was observed in contrast to pH 7.4 * releasing of RB remained low at both pH 5.5 and 7.4 *↑ ^1^O_2_ production by NPs in acidic than in neutral environment * sufficient ^1^O_2_ production by encapsulated RB *↑ intracellular accumulation and cytotoxicity of encapsulated DOX/RB in comparison to free solution * synergistic effect of encapsulated DOX/RB was detected	[52]
human lung adenocarcinoma (A549), cisplatin resistant human lung adenocarcinoma (A549R)	Ce6/cisplatin		MSNs	660 nm; 10 mW/cm^2^; 5 min	* average size of NPs: 100 nm * CD-PEI polyamine moiety on the surface of NPs facilitates NPs releasing from lysosomes to cytoplasm * MSNs with Ce6 and cisplatin showed significantly ↑ cytotoxicity and intracellular accumulation in comparison to free cisplatin * synergistic effect of Ce6 and cisplatin in MSNs was detected * utilization of MSNs leads to bypassing the traditional way of cisplatin transport to the cell including hCTR1 * intracellular transport of MSNs is mediated by endocytosis	[53]
murine breast carcinoma (4T1); human renal epithelial cells (293T)	ICG/DOX		CD44 functionalized HMSNs	808 nm; 5 min	* average size of NPs: 170 nm * ↑ release of drug under acidic pH * almost no fluorescence of NPs in 293T cells and detected fluorescence in 4T1 cells * excellent targeting ability against CD44 * NPs showed good biosafety in the dark conditions * ↑ cytotoxicity in experimental groups treated with functionalized NPs in comparison to other experimental groups	[54]
human lung carcinoma (A549)	Ce6/DOX		MCM-41 type MSNs	660 nM; 10 mW/cm^2^; 2 min	* average size: 100 nm * combination of DOX/Ce6 led to ↑ intracellular accumulation of drugs * free DOX was prevalently located in cell nucleus and encapsulated DOX was prevalently detected in cytoplasm * PDT leads to endosome destruction and DOX releasing to cytoplasm * NPs showed good biosafety in the dark conditions * synergistic effect of encapsulated DOX/Ce6 was detected	[55]
human cervical adenocarcinoma (HeLa)	Ru complexes		MSNs: MSN-CL-Ru, MSN-CNO-Ru, MSN-TRI-Ru	350 nm; 2.58 J cm^−2^; 10 min	* average size: 64–90 nm * cytotoxicity of MSNs-Ru was not significant in comparison to free Ru complexes that could be associated with low loading efficiency of MSNs	[56]
human breast adenocarcinoma (MCF-7; MCF-7 TX400—P-gp overexpressing; MCF-7 MX100—ABCG2 overexpressing; MCF-7/VP—MRP1 overexpressing)	BPD		porphyrin-lipid nanovesicles	690 nm, 0–20 J/cm^2^, 10 W/cm^2^	* BPD loaded in NPs is no longer a substrate for ABCG2 and becomes a weaker substrate for P-gp * significantly ↑ BPD accumulation and intracellular retention in MCF-7 MX100 and MCF-7 TX400 cells was observed if NPs were used in comparison to experimental groups treated with free BPD	[57]
DOX resistant human breast adenocarcinoma (MCF-7/ADR)	RB/DOX		triple-responsive nanogels	550 nm, 50 mW/cm^2^; 8 min	* average size: 153.5–244.9 nm * decreased temperature, reducing pH and enzyme treating promotes DOX releasing from NPs * loading of RB and DOX ↓ cell viability after PDT	[43]
multidrug resistant human melanoma cells (MDA-MB-435/MDR)	Ce6		biodegradable nanocomplex HA-Arg-PEA from (HAC) and arginine based poly(ester amide)s (ARG-PEA)	660 nm with light dose of 3 J/cm^2^, 2 min	* Arg-PEA component facilitated the formation of Ce6 monomer with ↑ photosensitizing efficiency * HA component achieved targeted delivery in CD44 positive tumor cells * monomerization of Ce6 loaded in NPs was observed in acidic pH compared to free Ce6 and ↑ generation of ^1^O_2_ was detected *↑ photocytotoxicity after PDT in tumor cells treated with Ce6 loaded in NPs in comparison to free Ce6	[44]
drug resistant human ovarian serous adenocarcinoma (NCI/ADR-RES)	MB/DOX		aerosol OT (AOT)-alginate nanoparticles	665 nm; 2400 mJ/cm^2^	* encapsulation in NPs was able to overcome resistance mechanisms and ↑ the cytotoxicity in resistant tumor cells *↑ ROS production, if combined therapy with NPs was applied in comparison to single drug treatment * combined therapy with NPs was able to overcome resistance mechanisms and resulted in ↑ cytotoxicity in drug-resistant tumor cells	[45]
MCF-7;MCF-7/ADR; female athymic nude mice	Ce6/DOX		inorganic ceria (cerium oxide NPs) nanocomposites	600 nm; 100 mW/cm^2^, 3 min	* NPs functionalized with FA ↑ cellular uptake of Ce6 * Ce6 loaded in NPs selectively accumulated in lysosomes and triggered ROS production after PDT *↓ expression of P-gp after PDT with Ce6 loaded in NPs detected *↑ chemotherapeutic efficacy of DOX and ↑ phototoxicity in drug-resistant cancer cells detected * apoptosis, autophagy and oncosis detected after PDT * significant tumor targeting and tumor growth inhibition observed	[46]
human cervical adenocarcinoma (HeLa), human ovarian serous cystadenocarcinoma (SKOV-3 and multidrug resistant SKOV-3/MDR); murine experimental model	NIR fluorophore (DEB-BDTO)/polymeric prodrug (PMP)		DEB/TQR@PMP	-------	* NPs exhibit synergistic effect of PDT and chemotherapy upon light irradiation to all 3 cell lines * in the tumor bearing mouse model, the DEB/TQR@PMP preferentially accumulated in the tumor tissue and overcame MDR and displayed ↑ inhibition of the tumor growth	[47]
DOX resistant human breast adenocarcinoma (MCF-7/ADR)	mitoxantrone		MIT-PFP/PPP	660 nm, 6, 12, 24 mW, 30 min	*↑ROS production, ↓ P-gp activity and ↑ cellular uptake of mitoxantrone after PDT MIT-PFP/PPP * after PDT MIT-PFP/PPP were able to ↑ ROS level, ↓ P-gp activity and ↑ cellular uptake of mitoxantrone * apoptotic cell death detected * reversed MDR detected	[48]
DOX resistant human breast adenocarcinoma (MCF-7/ADR); murine experimental model	disulfonated meso-tetraphenylporphine (TPPS2a)/DOX		IF7-ROSPCNP	10 J/cm^2^	* NPs underwent a dramatic structure disruption after exposing to a certain intensity of laser and then released free DOX *↑ cellular uptake of TPPS2a and DOX mediated by IF7 (specifically binds to annexin 1) improved cytotoxicity to tumor cells *↑ antitumor activity after functionalization with IF7	[49]

*, particular information related to MDR; ↑, means increase in observed parameter; ↓, means decrease in observed parameter; -------, the parameter was not provided by the authors; 5ALA, 5-aminolevulinic acid; BPD, benzoporphyrin derivative; Ce6, chlorin e6; DEB/TQR@PMP, DEB-BDTO/tariquidar and polymeric prodrug micelles; DOX, doxorubicin; FA, folic acid; HAC, hyaluronic acid; HB, hypocrellin B; ICG, indocyanine green; MIT-PFP-PPP, MIT-poly(ε-caprolactone)-pluronic F68-poly(ε-caprolactone)/poly(D,L-lactide-co-glycolide)–poly(ethylene glycol)–poly(D,L-lactide-co-glycolide); PMP, polymeric prodrug; RB, rose bengal; Ru, ruthenium; TPPS2a, disulfonated meso-tetraphenylporphine.

## Abbreviations

5ALA	5-aminolevulinic acid
ABC transporters	ATB-binding casette transporters
AlPcS_4_Cl	phthalocyanine chloride tetra sulfonic acid
BCRP/ABCG2	breast cancer resistant protein
BPD	benzoporphyrin derivative
BPD-MA	benzoporphyrin derivative monoacid ring A
CD-PEI	beta-cyclodextrin-grafted branched polyethylenimine
Ce6	chlorin e6
CSC	cancer stem cell
DEB/TQR@PMP	DEB-BDTO/tariquidar and polymeric prodrug micelles
DMSO	dimethyl sulfoxide
DNP	dynamic nanoplatform
DOX	doxorubicin
EPR	enhanced permeability and retention effect
FA	folic acid
FDA	Food and Drug Administration
HA	hypocrellin A
HAC	hyaluronic acid
HB	hypocrellin B
hCTR1	human copper transporter protein 1
HMSNPs	hollow-type mesoporous silica NPs
HPPH	2-[1-hexyloxyethyl]-2-devinyl pyropheophorbide-a
HY-PDT	photodynamic therapy with hypericin
ICG	indocyanine green
MB	methylene blue
MDR	multidrug resistance
MIT-PFP-PPP	MIT-poly(ε-caprolactone)-pluronic F68-poly(ε-caprolactone)/poly(D,L-lactide-co-glycolide)–poly(ethylene glycol)–poly(D,L-lactide-co-glycolide)
MRP1/ABCC1	MDR-associated protein-1
MSNs	nesoporous silica nanoparticles
mTHPC	meso-tetra-hydroxyphenyl-chlorin
NPs	nanoparticles
NS	nanosystem
PARP	poly (ADP-ribose) polymerase
Pc4	silicon phthalocyanine
PDT	photodynamic therapy
PEG	polyethylene glycol
P-gp/ABCB1	P-glycoprotein
PHPP	2,7,12,18-Tetra-methyl-3,8-di-(1-propoxyethyl)-13,17-bis-(3-hydroxypropyl) porphyrin
PLA	polyactic acid polymeric nanoparticles
PLGA	paclitaxel-loaded poly(DL-lactide-co-glycolide)
PMP	polymeric prodrug
PpIX	protoporphyrin IX
PSs	photosensitizers
PT	photofrin
RB	rose bengal
ROS	reactive oxygen species
Ru	ruthenium
SiPcCl_2_	silicon phthalocyanine dichloride
SLNs	solid lipid NPs
SP	side population
TPPS2a	disulfonated meso-tetraphenylporphine
TQR	tariquidar
ZnO	zinc oxide
